# Spatial networks of China's specialized, refined, distinctive, and innovative medical device firms based on parent–subsidiary contacts: implications for regional health policy

**DOI:** 10.3389/fpubh.2025.1676189

**Published:** 2025-11-26

**Authors:** Feng Hu, Huijie Yang, Shaobin Wei, Hao Hu, Yufeng Chen, Haiyan Zhou

**Affiliations:** 1 Institute of International Business & Economics Innovation and Governance, Shanghai University of International Business and Economics, Shanghai, China; 2 Inamori Business School, Zhejiang Gongshang University, Hangzhou, China; 3 International Business School, Shanghai University of International Business and Economics, Shanghai, China; 4 Institute of Digital Economy and Financial Powerhouse Building, Guangdong University of Finance, Guangzhou, China; 5 School of Economics, Shanghai University, Shanghai, China; 6 School of Economics and Management, Zhejiang Normal University, Jinhua, China; 7 Graduate School, Nueva Ecija University of Science and Technology, Cabanatuan, Philippines

**Keywords:** medical equipment manufacturer, social network analysis, network structure, health policy, innovation networks, parent–subsidiary relationships

## Abstract

This study examines the spatial structure and determinants of China's specialized, refined, distinctive, and innovative (SRDI) medical device industry through parent–subsidiary linkages. By combining social network analysis and geographical detectors, we analyze nationwide firm-level data from Qixinbao to explore network connectivity, centrality, and their socioeconomic drivers. The results show that (1) the overall network connectivity among Chinese cities is weak, with clear east–west disparities and Shanghai, Guangzhou, Beijing, and Shenzhen functioning as core hubs; (2) four distinct network subgroups exist, each assuming different roles in information and resource diffusion; and (3) factors such as economic base, degree of openness, and technological innovation are strongly associated with variations in network centrality across cities. These findings highlight the uneven spatial organization of China's SRDI medical device industry and underline the importance of strengthening interregional linkages, supporting innovation ecosystems, and improving talent and trade openness in less-developed regions. The study contributes to the literature by combining network analysis with spatial heterogeneity detection, offering empirical evidence for more balanced regional health industry development.

## 1 Introduction

As the main body of the real economy, small and medium-sized enterprises (SMEs) are the core actors of economic and social development (1, 2). Specialized, refined, distinctive, and innovative (SRDI) SMEs refer to those SMEs that are specialized, unique, and innovative, and this concept is similar to that of “hidden champion” enterprises in developed countries. As industry leaders, these companies are not well known and have sales revenues of less than a billion dollars, but they often rank in the top three nationally in terms of market share and are the cornerstone of the country's economy and trade (3). China's SRDI strategy was proposed by the Ministry of Industry and Information Technology in 2011 and appeared in the 2022 Chinese government work report; the development of SRDI SMEs can enhance not only China's discourse rights in the global industrial chain but also its resilience in terms of economic development. Therefore, what is the network structure of Chinese SRDI medical device manufacturing firms? What are the differences between this structure and other structures? What are the influencing factors of this network? Scientific network analysis methods are needed for comprehensive deconstruction.

Against the background that contemporary economic geography studies emphasize “space of flows,” academia has gradually focused on the structure of cyberspace. SMEs are the driving force behind regional economic development and play a crucial role in creating employment opportunities and enhancing people's livelihoods. Therefore, interfirm networks have become a research hotspot (4–6). Interfirm mergers and acquisitions (M&As), investment deals, and parent-subsidiary contacts form the space of flows across firms with different attributes (7, 8). A strong parent company controls subsidiary companies through capital, personnel, and technology. Parent-subsidiary contacts, which play a central role in business networks, can inevitably drive the flow of factors between cities when parent and subsidiary companies are located in different cities, thereby impacting economic development. Therefore, investigating the network structure of China's SRDI medical device manufacturing firms is very important (9–11). Weak or unevenly distributed networks can exacerbate regional disparities in healthcare innovation, while strengthening these linkages can facilitate the equitable allocation of medical technologies and support the development of resilient health systems. Therefore, understanding these parent-subsidiary networks is vital for designing industrial and health policies that foster innovation ecosystems, reduce geographic inequities, and enhance public health outcomes.

For the study of the parent-subsidiary relationship network, the Globalization and World Cities (GaWC) research network, represented by Taylor and Derudder et al., first used the chain network model to study the global city network based on the distribution data of the parent and subsidiary companies (12–15). Rozenblat et al. (16) analyzed the top 300 European multinational enterprises based on parent-subsidiary relationships and studied the spatiotemporal characteristics of European city networks. Based on the distribution data of the subsidiary companies of the world's top 500 enterprises, Alderson et al. (17) explored the attribute structure of the network. Based on the data of executives, Jin found that the larger the network is, the greater the efficiency of interfirm communication and the weaker the financing constraints faced by the firm (18). Moreover, Mehdi Khedmati et al. (19) investigated the relationship between the closeness of network contacts and the efficiency of labor investment by constructing network relationships between the chief executive officer (CEO) and board members. Based on the parent-subsidiary data of Fortune 500 companies and China's top 500 listed companies, Yang, Sheng, Wang et al. explored the spatial characteristics and influencing mechanisms of China's urban network (20–22). Some scholars have also started by considering specific industries, such as the automobile, finance, and logistics industries (23–25). For example, Wall et al., (26) based on data of the world's 100 largest multinational enterprises and their branches, found that the networks of advanced producer services (APS) enterprises and the industry-wide enterprises are very similar.

Previous studies on medical device industries have mainly focused on firms' development strategies (27–30), marketing management strategies (31–33), and supply chain strategies (34–37), with limited attention to SRDI firms—a rapidly growing but structurally underexplored segment. This gap is particularly relevant in the context of China's medical equipment manufacturing industry, which still lags behind developed countries in terms of enterprise scale, industry concentration, technological innovation, and international competitiveness. Moreover, most existing research relies on linear statistical models and pays little attention to the spatial network structure that underpins intercity linkages in the industry. This gap is particularly critical because SRDI firms often form complex parent–subsidiary networks that shape regional innovation flows and industrial clustering. To address this gap, this study integrates social network analysis and geographical detectors to analyze the spatial structure and socioeconomic determinants of the SRDI medical device industry in China, offering a novel methodological approach and new empirical insights.

Given the above factors, this study uses the parent–subsidiary contact data of SRDI medical device manufacturing firms to examine their spatial network morphology, which helps clarify the multilateral connections among Chinese medical equipment manufacturers and provides empirical evidence to guide the efficient allocation of resources, not only across enterprises but also within regional healthcare systems. In addition, this paper analyzes the determinants of the network to offer policy-oriented insights for optimizing the development and spatial planning of SRDI medical device manufacturing clusters, intending to support equitable access to medical technologies and strengthen regional health system resilience.

## 2 Research data and method

### 2.1 Research data

The initial firm-level data for SRDI medical device manufacturers were extracted from the Qixinbao database in November 2023. This database is updated synchronously with the website of the State Administration for Industry and Commerce of the People's Republic of China. To ensure the reliability of the research data, this study uses the national and provincial directories of specialized, refined, distinctive, and innovative enterprises for calibration. The data of the subsidiaries of these medical equipment manufacturers are obtained from the corporate affiliate board of Qixinbao, through which the registered addresses and main industry types of the subsidiaries are sorted. An intercity directed multivalued network matrix is formed based on the contacts between the cities where the parent and subsidiary companies are located.

### 2.2 Research methods

#### 2.2.1 Matrix construction method

We constructed a directed, weighted intercity network matrix. The contact strength D (m, n) between cities m and n reflects the network contact strength between the two cities established through all parent-subsidiary contacts, the calculation formula of which is as follows:


$$\begin{array}{l} D\;\left(m,n\right)=\overset{a}{\underset{\text{i}=1}{\sum }}{\text{v}}_{\text{im}}v_{in} \end{array}$$

where *a* represents the number of enterprises that have established parent or subsidiary companies in both cities *m* and *n*. V_im_ denotes the number of branches of firm *i* in city *m*. V_in_ denotes the number of branches of firm *i* in city *n*. Each city pair's contact strength D (m, n) is then assigned to the corresponding element in the network matrix D. To illustrate, consider two cities, m and n, and two enterprises, A and B, each with branches in both cities. Firm A has two branches in city m and one branch in city n. B has three branches in m and two branches in n, respectively. Thus, v_am_ is 2, v_an_ is 1, v_bm_ is 3, and v_bn_ is 2. The contact strength between m and n is v_am_ v_an_ + v_bm_ v_bn_ = 8. This value is then entered into the corresponding position in the matrix D, representing the overall network contact strength between the two cities.

#### 2.2.2 Social network analysis

Social network analysis is used to analyze the changes in the centrality, network density, and cohesive subgroups of the network of Chinese SRDI medical device manufacturing firms and reveal the characteristics of the node cities in the network, such as their network location and importance, the closeness of their network contacts, and the relationship pattern between cities (38–42).

#### 2.2.3 Geographical detectors (geodetectors)

The geodetector factor detection model is used to study the factors influencing the formation of the specialized, unique, and innovative medical equipment manufacturer network in China. Compared with traditional regression or spatial econometric models, Geodetector offers several advantages: it can capture nonlinear relationships, account for spatial heterogeneity by considering the stratification of variables typical in regional industrial networks, detect interactions between factors—whether they act independently, reinforce, or weaken each other's effects—and remain robust to multicollinearity, so that highly correlated factors do not bias the assessment of their individual explanatory power. The analysis was implemented in the official standalone Geodetector software. For the calculation formula of the factor detection module, please refer to references (43–47).

## 3 Overall characteristics

### 3.1 Weak parent-subsidiary network contact, with Shanghai and Guangzhou serving as the main radiation-driven centers

**Figure 1:**
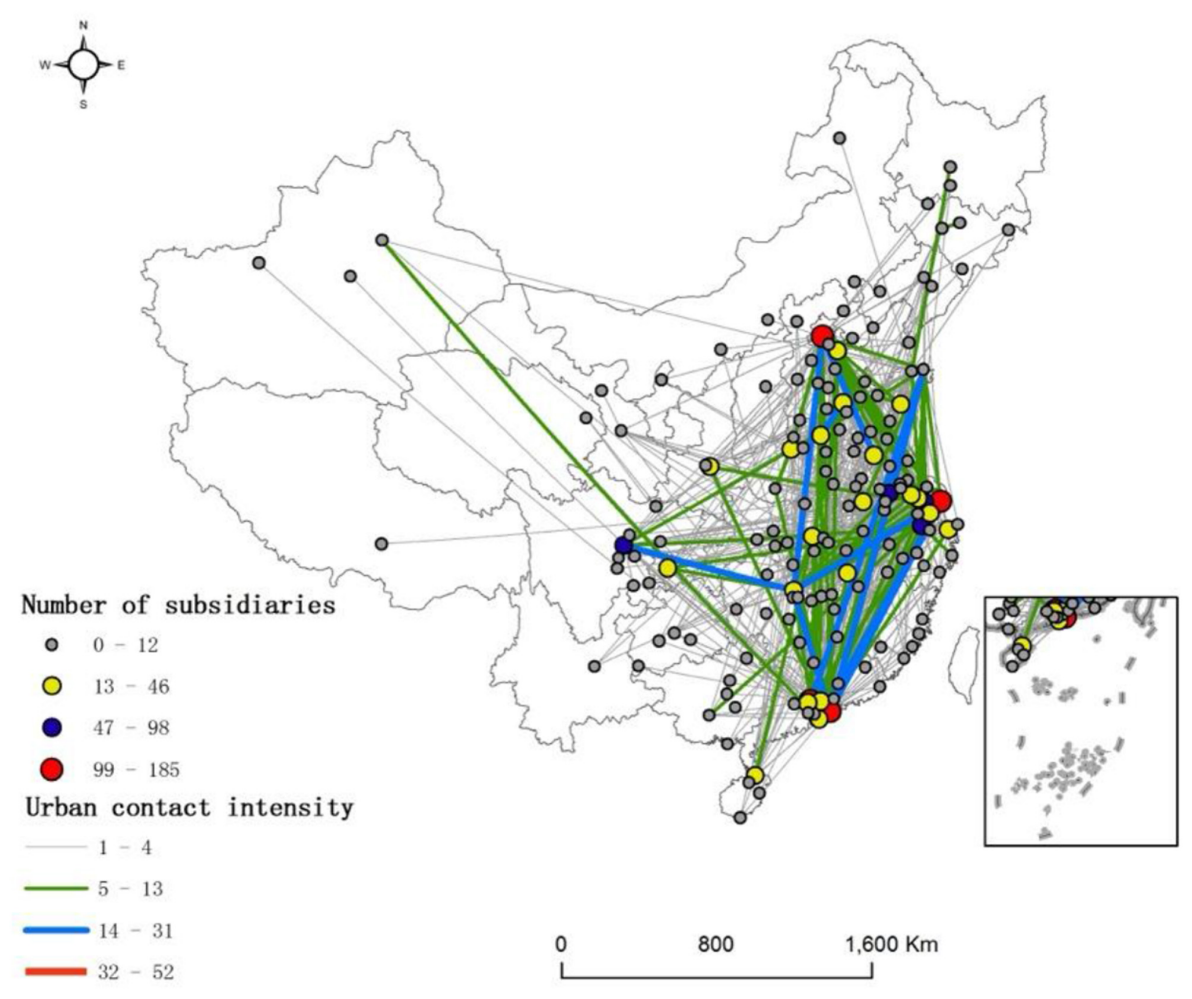
Number and contact network of the subsidiaries of SRDI medical device manufacturing firms in each city in China.

As shown in Figure 1, nearly 70% of the cities in China are included in the network of China's SRDI medical device manufacturing firms, but the network density is only 0.025, indicating that the overall contact level is relatively weak. There are 675 contact edges between cities, and the average contact strength is 10.78. The overall network structure is a multicenter radial network structure, with Shanghai, Guangzhou, Beijing, and Shenzhen as the main radiation-driven centers and Hangzhou, Suzhou, Chengdu, and Nanjing as the main nodes. Specifically, the network density is the highest in the eastern coastal region, followed by the central region, and then the western region. The cities in the central and eastern regions are closely connected, and the subsidiaries of China's SRDI medical device manufacturing firms are concentrated mainly in the Guangdong-Hong Kong-Macao Greater Bay Area, the Yangtze River Delta urban agglomeration, and the Beijing-Tianjin-Hebei urban agglomeration.

### 3.2 Heterogeneity of internal and external contacts in the eastern, central, and western regions

According to Table 1, both internal and external contacts in the eastern region are strong, indicating that the eastern region is the region where SRDI medical device manufacturing firms in China are most closely connected. There is a large gap between the central and western regions and the eastern region in terms of both internal and external contacts. Especially in terms of internal contacts, the central and western regions account for only 5.88 and 1.04%, respectively, of those of the eastern region. The proportions of parent cities show that the internal contact of the eastern region and the external contact of the central region account for the largest proportion, while the internal contact of the western region and the external contact of the eastern region account for the smallest proportion, indicating that for the internal contacts in the eastern region, the parent city, as the radiation-driven center, is the dominant city, and for the external contacts, multicenter radiation is more prevalent. In the central and western regions, only a few cities play the role of contact with medical equipment manufacturers. In general, the contacts among China's SRDI medical device manufacturing firms exhibit a pattern in which there is denseness in the east and sparseness in the west.

**Table 1:** Characteristics of the strength of internal and external ties and the first-place share in the Eastern, Central, and Western regions of China.

**Region**	**Strength of intraregional linkages**		**Strength of regional external linkages**	
	**Contact strength**	**Top quartile**	**Contact strength**	**Top quartile**
Eastern region	1,053	0.12	353.00	0.14
Central region	62	0.08	234.00	0.67
Western region	11	0.06	55.00	0.40

### 3.3 Differential input and output of network subgroups

To identify cohesive subgroups within the intercity network, we employed the CONCOR algorithm using UCINET 6 for Windows. The analysis was configured with a maximum of 25 iterations and a convergence criterion of 0.200. The network was recursively partitioned to a maximum depth of four splits, resulting in a final structure of meaningful subgroups for interpretation. Table 2 shows four subgroups for the network of SRDI medical device manufacturing firms in China. Of these four subgroups, the second subgroup has the largest number of members. It is worth noting that the division of subgroups does not strictly adhere to the principle of geographic proximity. For instance, Shanghai and Nanjing, both located within the Yangtze River Delta, are assigned to different subgroups. This suggests that the structure of corporate networks may be driven more by a city's functional positioning and industrial complementarity than by mere geographic distance.

**Table 2:** Subgroups of China's SRDI medical device manufacturing firms networks.

**Subgroup**	**Subgroup member**
1	Zhongshan, Heyuan, Nanjing, Xiamen, Suzhou and 36 other cities
2	Shenzhen, Tianjing, Changsha, Shanghai, Zhuhai and 51 other cities
3	Weihai, Huizhou, Lishui, Tangshan, Yueyang and 38 other cities
4	Liuzhou, Bozhou, Dongguan, Fuzhou, Guigang and 39 other cities

Table 3 shows that each subgroup plays a different role in the network of SRDI medical device manufacturing firms. The main output direction of subgroup 1 is to subgroup 3 (density of 0.250). The main output direction of subgroups 4 and 2 is to subgroup 1 (densities of 0.390 and 0.343, respectively). Subgroup 1 belongs to the beneficiary subgroup in the network, while subgroup 3 is marginal. The frequency of the internal contacts between subgroups 2 and 1 is at a high level.

**Table 3:** Density matrix and image matrix of subgroups of the network of SRDI medical device manufacturing firms in China.

**Subgroup**	**Density matrix**				**Image matrix**			
	**1**	**2**	**3**	**4**	**1**	**2**	**3**	**4**
1	0.207	0.216	0.250	0.000	1	1	1	0
2	0.343	0.215	0.068	0.091	1	1	0	0
3	0.087	0.000	0.000	0.000	0	0	0	0
4	0.390	0.148	0.000	0.000	1	1	0	0

### 3.4 Degrees of weighted centrality and diffusion of important cities

According to Table 4, Beijing, Zhuhai, Shenzhen, Shanghai, and Changsha are the core cities in terms of the deconstruction of the network of China's SRDI medical device manufacturing firms, mainly because these cities are the main concentration areas of the parent and subsidiary companies of these manufacturers. Beijing, leveraging its absolute political and economic advantages, emerges as the primary core of the network, with a weighted centrality of 300. Notably, Zhuhai and Shenzhen—as regional innovation hubs—exceed even Shanghai (233) in centrality, which are 238 and 237, respectively, despite Shanghai's larger economic scale. This suggests that within specialized high-tech manufacturing networks, innovation clustering effects can sometimes surpass traditional economies of scale. Cities like Guangzhou and Hangzhou function as vital secondary nodes, receiving spillover effects from core cities. In these cities, the proportion of parent companies is close to 20%, while the proportion of subsidiaries is close to 30%. Zhuhai ranks first in terms of the degree of diffusion of parent and subsidiary companies, indicating that the city's SRDI medical device manufacturing firms have strong output ability but insufficient attraction ability. The degrees of diffusion of Shenzhen, Jiaxing, Xi'an, Heze, and Zhengzhou are approximately between −3 and 4, indicating that these cities are equivalent in terms of their ability to control and attract SRDI medical device manufacturing firms. Although Guangzhou, Chengdu, and Wuhan are among the top 20 cities in China in terms of weighted centrality, their degrees of diffusion are negative, indicating that their ability to attract the subsidiaries of SRDI medical device manufacturing firms is greater than is their control ability, thus causing them to assume the role of input core nodes in the network.

**Table 4:** Network node centrality and diffusivity of SRDI medical device manufacturing firms in China.

**City**	**Weighted centrality**	**Diffusivity**	**City**	**Weighted centrality**	**Diffusivity**
Beijing	300	26	Jinan	90	6
Zhuhai	238	38	Yantai	71	27
Shenzhen	237	4	Chengdu	68	−18
Shanghai	233	−7	Nanjing	67	−6
Changsha	226	12	Changzhou	62	16
Guangzhou	185	−10	Wuhan	46	−12
Hangzhou	161	7	Jiaxing	44	0
Weihai	152	27	Xian	43	−3
Suzhou	151	8	Heze	42	0
Tianjing	133	29	Zhengzhou	40	−1

### 3.5 Distribution pattern of the network of SRDI medical device manufacturing firms in China

To analyze in depth the spatial characteristics of the network of China's SRDI medical device manufacturing firms, a breadth-depth two-dimensional (2D) matrix is constructed. The breadth denotes the network centrality, that is, the number of other cities connected with a particular city, and the depth denotes the network weight, that is, the number of contacts of a particular city with other cities. The average breadth and depth are used as thresholds, and the network of China's SRDI medical device manufacturing firms can be divided into four types, i.e., high breadth-high depth (HH), high breadth-low depth (HL), low breadth-high depth (LH), and low breadth-low depth (LL) networks.

**Figure 2:**
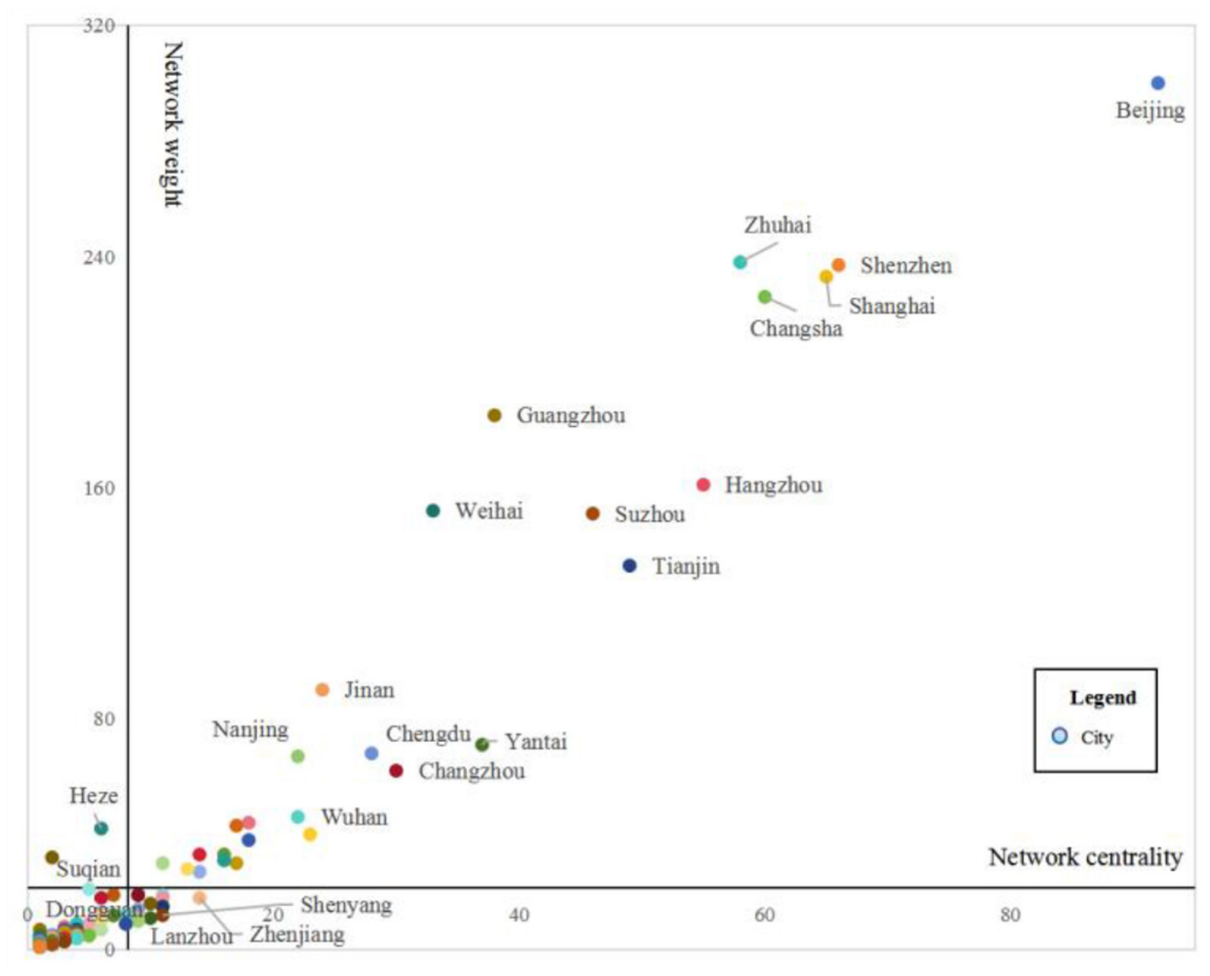
Degree distribution pattern of the network of China's SRDI medical device manufacturing firms.

As shown in Figure 2, there are a total of 27 cities in the HH quadrant, among which Beijing stands out and has contacts with more than half of the cities in the network. Cities such as Shanghai, Zhuhai, Shenzhen, and Changsha exceed other cities in terms of contact breadth and depth. There are 13 cities in the HL quadrant, including Zhenjiang, Shenyang, and Lanzhou, and the parent-subsidiary contact breadth of the SRDI medical device manufacturing firms in these cities exceeds that in other cities, but the contact depth is lower than the average contact depth across all cities. There are only two cities in the LH quadrant, namely, Heze and Suqian. These two cities have a relatively wide contact breadth with other cities, but their contact depth is insufficient. There are 122 cities in the LL quadrant. In the parent-subsidiary contacts of SRDI medical device manufacturing firms, most cities are below the average level in terms of contact depth and breadth.

### 3.6 Differences in the cyberspace structure of subsidiary companies in different industries

Since the subsidiaries of medical equipment manufacturers are involved in many industries and the business types of different subsidiaries are quite different, this paper further discusses the influence of different industry types. With reference to industry classification standards, the primary industries with the largest number of parent-subsidiary contacts are selected, i.e., scientific research and technology services (number of contacts: 804), manufacturing (number of contacts: 311), and wholesale and retail (number of contacts: 294).

According to Appendix Table 5, for the subsidiaries of China's SRDI medical device manufacturing firms in the scientific research and technology services industry, there is a network pattern with Beijing, Changsha, Zhuhai, Shanghai, and Guangzhou as the centers. These types of subsidiaries are more likely to be located in high-level cities, showing the characteristics of a spatial structure dominated by long-range radiation. The reason for this is that these cities have high levels of science and technology and a larger number of research institutes than do other cities.

For the subsidiaries of China's SRDI medical device manufacturing firms in the manufacturing industry, there is a network pattern with Shenzhen and Changsha as the centers, presenting a dual-core radial spatial pattern, with the spatial characteristics of overlapping short-range and long-distance radiation. The short-range radiation directions include Zhuhai → Guangzhou, Zhuhai → Shenzhen, and Shenzhen → Dongguan, and the long-distance radiation directions include Jinan → Haikou, Weihai → Shenzhen, and Changsha → Beijing.

For the subsidiaries of China's SRDI medical device manufacturing firms in the wholesale and retail industry, there is a network pattern with Shenzhen and Shanghai as the centers, and the location selection of these types of subsidiaries is more inclined toward cities with high levels of market demand. The cities with high-level contact strength are Jinan → Heze, Suzhou → Shanghai, Tianjin → Guangzhou, Zhuhai → Shenzhen, and Weihai → Shenzhen. Furthermore, Shenzhen, Shanghai, Guangzhou, and other cities have large resident populations and abundant medical resources; thus, their demand for medical devices is at a relatively high level, and these cities generally also serve as distributed production centers for medical devices.

## 4 Analysis of influencing factors

### 4.1 Selection of influencing factors

According to previous studies (36–38), in this study, the network weighted centrality is used as the explained variable, the gross domestic product (GDP), the average wage of employees, the total amount of imports and exports, the number of colleges and universities, telecom service income, the number of patent authorizations, the number of hospital beds, and the urban administrative level are selected as influencing factors, and geodetectors are used to analyze the correlation coefficients between the network weighted centrality and its influencing factors. For the Geodetector analysis, all continuous independent variables were discretized using the “natural breaks” method in ArcGIS Pro into five strata. This method was selected as it minimizes variance within classes and maximizes variance between classes, making it suitable for detecting spatial heterogeneity. The administrative level variable was treated as a categorical variable. For the administrative level, Beijing is set to 5; Shanghai, Chongqing, and Tianjin are set to 4; provincial capitals are set to 3; subprovincial cities are set to 2; and general cities are set to 1. We subsequently used the factor detector module to calculate the Q-statistic, which measures the proportion of the weighted centrality's spatial variance explained by each factor.

### 4.2 Result analysis

The geodetector results (Appendix Table 6) reveal that the spatial heterogeneity of the weighted centrality in the urban network is significantly associated with the seven selected factors. This suggests that cities with advantages in economic base, labor wage level, degree of openness, talent scale, technological innovation, market demand, and political resources tend to exhibit higher network centrality. The explanatory power (Q-value) of each factor ranges between 20 and 60%.

Among these factors, economic foundation, degree of openness, and technological innovation demonstrate the strongest associations with the network structure of China's SRDI medical device manufacturing firms, consistently ranking in the top three across all network specifications, including those with and without different subsidiaries, both with and without different types of subsidiaries. This finding aligns strongly with our theoretical expectation that robust GDP provides the essential market and capital for enterprise establishment and expansion, while active patenting activity signifies regional innovation vitality, both critical for the location choice of technology-intensive firms like SRDI.

Wage level, talent scale, and market demand are also significantly correlated with the network structure. The positive correlation between wage level and centrality may reflect the co-location of higher-paying cities and a more qualified labor force. Notably, the stronger correlation observed for subsidiaries in the scientific research and technology services industry suggests that the operations in this sector might be more sensitive to the local availability of a specialized professional workforce. Furthermore, the positive association with the number of higher education institutions underscores their potential role as key nodes, possibly through industry-university-research cooperation in fostering innovation for the medical equipment sector. Market demand, proxied by the number of hospital beds, also shows a significant statistical relationship with the firm network.

The association between political resources and the overall firm network is relatively weak, with a Q-value of 0.2797 and marginal significance at the 10% level. For the specific industries of scientific research and technology services, manufacturing, and wholesale and retail, the Q-values for political resources are above 0.2 but are not statistically significant. This pattern suggests that while higher-level administrative cities, presumably with richer political and informational resources, are associated with a greater presence of SRDI firms in the aggregate network, this relationship is not a robust or consistent determinant across specific sub-sectors.

## 5 Discussion and conclusions

### 5.1 Conclusions

In this study, using the data of SRDI medical device manufacturing firms in China, from the perspective of parent-subsidiary contacts, the network structure is studied through social network analysis and geodetectors. The following conclusions are obtained.

(1) Overall, the network contacts of China's SRDI medical device manufacturing firms are relatively weak, which may limit the diffusion of advanced medical technologies, potentially exacerbating regional inequities in healthcare accessibility. With Shanghai, Guangzhou, and Beijing as the centers, the subsidiaries are concentrated mainly in the Guangdong-Hong Kong-Macao Greater Bay Area, the Yangtze River Delta urban agglomeration area, and the Beijing-Tianjin-Hebei urban agglomeration area. Furthermore, the internal and external contacts of the eastern region are greater than those of the central and western regions.
(2) The network has four subgroups. Subgroups 1, 2, and 4 play the role of outputs in the network of China's SRDI medical device manufacturing firms, with their inputs and outputs being different. In contrast, Beijing, Zhuhai, Shenzhen, Shanghai, and Changsha are the agglomeration and diffusion centers of the network of China's SRDI medical device manufacturing firms.
(3) Cities such as Shanghai, Zhuhai, Shenzhen, and Changsha surpass other cities in terms of contact breadth and depth. Most cities are edge cities, the contact breadth and depth of which are below the average level of all cities in China. The network of China's SRDI medical device manufacturing firms, whose subsidiaries are in the scientific research and technology services industry, includes Beijing, Changsha, Zhuhai, Shanghai, and Guangzhou as its centers. The network of China's SRDI medical device manufacturing firms, whose subsidiaries are in the manufacturing industry, includes Shenzhen and Changsha as its centers, presenting a dual-core radial spatial pattern. The network of China's SRDI medical device manufacturing firms, whose subsidiaries are in the wholesale and retail industry, includes Shenzhen and Shanghai as its centers.
(4) Seven factors, namely, economic base, wage level, degree of openness, talent scale, technological innovation, market demand, and political resources, can affect the network morphology of SRDI medical device manufacturing firms. Addressing these determinants through policy measures can simultaneously enhance industrial competitiveness and strengthen the healthcare system's capacity to deliver innovative, timely, and equitable care across China.

### 5.2 Policy implications

(1) Based on the findings of this study, we propose a tripartite policy framework aimed at optimizing the SRDI medical device manufacturing network, with a specific focus on mitigating regional disparities and enhancing the resilience of the national healthcare innovation system. First, policy must foster strategic linkages between core and peripheral regions through targeted interventions. While established hubs like Beijing, Shanghai, and Shenzhen should continue to drive high-end innovation, provincial and municipal governments should incentivize them to establish technology transfer programs and R&D partnerships with firms in less-developed regions. This can be achieved through mechanisms such as targeted subsidies administered by provincial health commissions and tax credits for core enterprises that invest in or mentor SRDI firms in central and western cities. Concurrently, underdeveloped cities (e.g., Shantou, Changde, and Wuhu) must improve their absorptive capacity by optimizing local business environments and investing in specialized industrial parks.
(2) Enhancing regional innovation ecosystems requires a concerted effort to bolster talent pools, technological capability, and market openness. Municipal science and technology bureaus and human resources departments should launch specialized talent programs. Furthermore, local governments should increase R&D spending through municipal innovation funds specifically earmarked for medical device prototyping and clinical trials. To integrate local firms into global value chains, the Ministry of Commerce and industry associations could facilitate international collaboration channels and streamline regulatory processes for exports. These actions directly address the strong association of talent, innovation, and openness with network centrality revealed by our analysis.
(3) A national-level coordinated strategy is essential to systematically reduce interregional fragmentation and build a more resilient health industry landscape. We recommend that the National Development and Reform Commission (NDRC) and the National Health Commission (NHC) take the lead in establishing a cross-regional medical device industry alliance, coordinating resource sharing, joint procurement of raw materials, and mutual recognition of quality standards. Additionally, an infrastructure plan should be enacted to develop dedicated freight and data corridors connecting eastern innovation hubs with industrial parks in the central and western regions. By specifying responsible actors and practical mechanisms, these recommendations provide a concrete roadmap for achieving a more balanced and robust medical device manufacturing network, which is fundamental to ensuring equitable access to medical technologies and strengthening public health system preparedness across China.

### 5.3 Theoretical contributions

The theoretical contributions of this study are reflected mainly in the following aspects.

First, this study is a powerful supplement to studies on the medical equipment manufacturing industry. Compared to those studies that investigate the development strategy (27–30), marketing management strategy (31–33), and supply chain strategy (34–37) of medical equipment manufacturers, this study focuses on the specialized, unique, and innovative medical equipment manufacturing industry, which not only fills the gap in the study of such manufacturers but also helps comprehensively explain the spatial network distribution of this industry.

Second, from a spatial network perspective, this study uses social network analysis to comprehensively analyze the spatial network morphology of the parent-subsidiary contacts of SRDI medical device manufacturing firms, which is helpful for promoting the further application and development of social network analysis methods. Moreover, this study can promote the development of regional industry theory, promote beneficial exploration for the injection of enterprise organization spatial structure and city status into industrial theory, and provide a direct reference for the layout of the medical equipment manufacturing industry (7–25).

Finally, different from studies on physical flows, such as population and trade flows, this study focuses on organizational flow, in soft information flow, and uses data on the space of flows. Moreover, based on the parent-subsidiary contact data of SRDI medical device manufacturing firms, this work studies the urban network structure, which acts as a supplement to traditional studies based on attribute data. The space of flows of specific industries can more accurately reflect the form and role of the intercity spatial network, thus further deepening and expanding the theory of space of flows (48–54).

### 5.4 Limitations and prospects for future research

This study investigates the network structure of SRDI medical device manufacturing firms from the perspective of parent-subsidiary contacts. However, the following limitations remain to be overcome. First, despite covering 70% of Chinese cities, our data may underrepresent rural areas, potentially limiting the generalizability of findings to regions with the most acute healthcare needs. The reliance on the Qixinbao database, without cross-validation, may also introduce coverage biases. Second, a key methodological limitation is the equal weighting of all subsidiaries, regardless of their size, investment, or employment. This assumption, dictated by data availability, may bias the network structure. Future work should employ investment or employment data for robustness checks. Third, our analysis focuses on organizational structure without incorporating direct measures of innovation output. Future research should integrate such data to better link network position with innovative performance. Lastly, the cross-sectional design prevents causal inference. Longitudinal analyses are needed to uncover the dynamic relationships between network evolution and its drivers.

## Data Availability

The raw data supporting the conclusions of this article will be made available by the authors, without undue reservation.
