# Supplementary material for: Spatial networks of China's specialized, refined, distinctive, and innovative medical device firms based on parent–subsidiary contacts: implications for regional health policy
Source: Front Public Health. 2025 Nov 26;13:1676189. doi: 10.3389/fpubh.2025.1676189 (PMC12689987; doi:10.3389/fpubh.2025.1676189)
Supplement: Supplementary file 1 [file Table_1.docx]

**Appendix 1 Table 5** City weighting and linkage pairs of the contact network of specialized and new medical device enterprises by industry (top twenty)

|  | **Scientific research and technical services** | | | | **Service industry** | | | | **Wholesale and retail trade** | | | |
| --- | --- | --- | --- | --- | --- | --- | --- | --- | --- | --- | --- | --- |
|  | City | weighting | couple | NOC | City | weighting | couple | NOC | City | weighting | couple | NOC |
| 1 | Beijing | 149 | Zhuhai→Guangzhou | 33 | Shenzhen | 52 | Changsha→Shenzhen | 8 | Shenzhen | 47 | Jinan→Heze | 13 |
| 2 | Changsha | 131 | Weihai→Guangzhou | 20 | Changsha | 45 | Jinan→Heze | 7 | Shanghai | 45 | Suzhou→Shanghai | 7 |
| 3 | Zhuhai | 123 | Tianjin→Beijing | 16 | Zhuhai | 33 | Heze→Jinan | 7 | Guangzhou | 32 | Tianjin→Guangzhou | 6 |
| 4 | Shanghai | 123 | Changsha→Beijing | 16 | Shanghai | 32 | Shenzhen→Dongguan | 5 | Changsha | 28 | Zhuhai→Shenzhen | 6 |
| 5 | Guangzhou | 114 | Zhuhai→Shanghai | 16 | Jinan | 30 | Jinan→Haikou | 5 | Yantai | 28 | Weihai→Shenzhen | 6 |
| 6 | Shenzhen | 97 | Shenzhen→Beijing | 11 | Beijing | 29 | Zhuhai→Guangzhou | 4 | Jinan | 27 | Heze→Jinan | 5 |
| 7 | Suzhou | 86 | Suzhou→Shanghai | 11 | Suzhou | 27 | Shenzhen→Xiatan | 4 | Zhuhai | 27 | Shenzhen→Shanghai | 4 |
| 8 | Weihai | 81 | Zhuhai→Beijing | 10 | Hangzhou | 25 | Zhuhai→Nanjing | 4 | Weihai | 27 | Zhuhai→Guangzhou | 4 |
| 9 | Hangzhou | 65 | Changsha→Nanjing | 10 | Weihai | 20 | Changsha→Beijing | 4 | Tianjin | 26 | Tianjin→Shenzhen | 4 |
| 10 | Tianjin | 62 | Zhuhai→Shenzhen | 9 | Heze | 16 | Suzhou→Shenzhen | 4 | Beijing | 26 | Changsha→Guangzhou | 4 |
| 11 | Nanjing | 34 | Changsha→Shanghai | 8 | Guangzhou | 16 | Zhuhai→Hangzhou | 3 | Suzhou | 20 | Changsha→Shanghai | 4 |
| 12 | Changzhou | 32 | Jiaxing→Shanghai | 8 | Nanjing | 13 | Taizhou→Hangzhou | 3 | Heze | 18 | Beijing→Taizhou | 3 |
| 13 | Chengdu | 27 | Xian→Dongguan | 8 | Tianjing | 13 | Suzhou→Suqian | 3 | Hangzhou | 14 | Nanning→Shanghai | 3 |
| 14 | Jinan | 27 | Shenzhen→Shanghai | 8 | Chengdu | 11 | Weihai→Zhen | 3 | Chengdu | 13 | Weihai→Shanghai | 3 |
| 15 | Wuxi | 25 | Weihai→Shanghai | 8 | Changzhou | 11 | Zhuhai→Shenzhen | 3 | Zhengzhou | 13 | Huzhou→Hangzhou | 3 |
| 16 | Wuhan | 24 | Chasha→Chengdu | 7 | Wuhan | 10 | Changsha→Chengdu | 3 | Ningbo | 10 | Changsha→Beijing | 3 |
| 17 | Xian | 22 | Shenzhen→Guangzhou | 7 | Yantai | 10 | Weihai→Nanjing | 3 | Xian | 8 | Guangzhou→Shanghai | 3 |
| 18 | Jiaxing | 20 | Weihai→Beijing | 7 | Dongguan | 8 | Shanghai→Changzhou | 2 | Huzhou | 7 | Changsha→Chengdu | 3 |
| 19 | Hefei | 17 | Weihai→Suzhou | 7 | Xian | 8 | Weifang→Qingdao | 2 | Nanchang | 7 | Weihai→Guangzhou | 3 |
| 20 | Zhengzhou | 16 | Guangzhou→Shanghai | 7 | Ningbo | 8 | Zhuhai→Chenzhou | 2 | Wuhan | 7 | Zibo→Shanghai | 3 |

Note: NOC is an abbreviation for Number of connections
