# Supplementary material for: Spatial networks of China's specialized, refined, distinctive, and innovative medical device firms based on parent–subsidiary contacts: implications for regional health policy
Source: Front Public Health. 2025 Nov 26;13:1676189. doi: 10.3389/fpubh.2025.1676189 (PMC12689987; doi:10.3389/fpubh.2025.1676189)
Supplement: Supplementary file 2 [file Table_2.docx]

**Appendix 2 Table 6** Geodetector analysis result

| **Factors** | **Measurement indicators** | **Network of Medical Device Manufacturers** | | **Scientific research and technical services** | | **Service industry** | | **Wholesale and retail trade** | |
| --- | --- | --- | --- | --- | --- | --- | --- | --- | --- |
|  |  | Q-value | Significance | Q-value | Significance | Q-value | Significance | Q-value | Significance |
| **Economic foundation** | GDP | 0.5971 | 0.000*** | 0.5789 | 0.000*** | 0.5188 | 0.000*** | 0.4765 | 0.000*** |
| **Wage level of labor force** | Urban non-private sector  Average wage of on-post workers | 0.4235 | 0.000*** | 0.4694 | 0.000*** | 0.4130 | 0.000*** | 0.3396 | 0.000*** |
| **Open to the outside world** | Total exports and imports | 0.5729 | 0.000*** | 0.5557 | 0.000*** | 0.5049 | 0.000*** | 0.4489 | 0.000*** |
| **Size of talent** | Number of general higher education institutions | 0.3591 | 0.000*** | 0.3742 | 0.000*** | 0.2857 | 0.000*** | 0.2914 | 0.000*** |
| **Technological innovation** | Number of patents granted | 0.5880 | 0.000*** | 0.6037 | 0.000*** | 0.5222 | 0.000*** | 0.4601 | 0.000*** |
| **Market** | Number of hospital beds | 0.4159 | 0.000*** | 0.4103 | 0.000*** | 0.3905 | 0.000*** | 0.3412 | 0.000*** |
| **Political resources** | Municipal administrative level | 0.2797 | 0.059* | 0.2947 | 0.110 | 0.2408 | 0.243 | 0.2734 | 0.144 |

Significance levels are indicated as follows: *** p ≤ 0.01 (highly significant), ** 0.01 < p ≤ 0.05 (significant), * 0.05 < p ≤ 0.10 (marginally significant), and no asterisk indicates p > 0.10 (not significant)
